# The role of the Smad2/3/4 signaling pathway in osteogenic differentiation regulation by ClC-3 chloride channels in MC3T3-E1 cells

**DOI:** 10.1186/s13018-022-03230-1

**Published:** 2022-07-06

**Authors:** Xiaolin Lu, Weixu Li, Huan Wang, Meng Cao, Zuolin Jin

**Affiliations:** 1 State Key Laboratory of Military Stomatology, National Clinical Research Center for Oral Diseases, Shaanxi Clinical Research Center for Oral Diseases, Fourth Military Medical University, Xi’an, 710032 China; 2 Department of Orthodontics, School of Stomatology, Fourth Military Medical University, Xi’an, 710032 China

**Keywords:** ClC-3 chloride channels, Smad2/3/4 signaling pathway, Nuclear translocation, Osteogenic differentiation, Bone metabolism

## Abstract

**Background:**

ClC-3 chloride channels promote osteogenic differentiation. Transforming growth factor-β1 (TGF-β1) and its receptors are closely related to ClC-3 chloride channels, and canonical TGF-β1 signaling is largely mediated by Smad proteins. The current study aimed to explore the role of the Smad2/3/4 signaling pathway in the mechanism by which ClC-3 chloride channels regulate osteogenic differentiation in osteoblasts.

**Methods:**

First, real-time PCR and western blotting were used to detect the expression of Smad and mitogen-activated protein kinase (MAPK) proteins in response to ClC-3 chloride channels. Second, immunocytochemistry, coimmunoprecipitation (Co-IP) and immunofluorescence analyses were conducted to assess formation of the Smad2/3/4 complex and its translocation to the nucleus. Finally, markers of osteogenic differentiation were determined by real-time PCR, western blotting, ALP assays and Alizarin Red S staining.

**Results:**

ClC-3 chloride channels knockdown led to increased expression of Smad2/3 but no significant change in p38 or Erk1/2. Furthermore, ClC-3 chloride channels knockdown resulted in increases in the formation of the Smad2/3/4 complex and its translocation to the nucleus. In contrast, the inhibition of TGF-β1 receptors decreased the expression of Smad2, Smad3, p38, and Erk1/2 and the formation of the Smad2/3/4 complex. Finally, the expression of osteogenesis-related markers were decreased upon ClC-3 and Smad2/3/4 knockdown, but the degree to which these parameters were altered was decreased upon the knockdown of ClC-3 and Smad2/3/4 together compared to independent knockdown of ClC-3 or Smad2/3/4.

**Conclusions:**

The Smad2/3 proteins respond to changes in ClC-3 chloride channels. The Smad2/3/4 signaling pathway inhibits osteogenic differentiation regulation by ClC-3 chloride channels in MC3T3-E1 cells.

## Introduction

Bone is a kind of highly mineralized tissue that is always under regulation by dynamic balance between the processes of bone formation and bone resorption. This homeostasis is mainly supported by the coordinated activities of osteoblasts and osteoclasts. The mechanism of bone metabolic regulation is extremely complex, and abnormalities in any step may lead to bone metabolic disorders, such as osteoporosis and osteopetrosis [1–3].

As one of the most abundant anions in organisms, chloride ions regulate a very wide range of physiological activities. The ClC-3 chloride channels, a kind of voltage-gated anion channel, participates in ion homeostasis, the regulation of cell volume, acidification of the internal environment, proliferation, apoptosis and many other processes in biological systems [4, 5]. Recent studies have found that ClC-3 chloride channels are closely related to bone metabolism. Clcn3-knockout mice displayed complex genetic phenotypes, including stunting, kyphosis, and abnormalities in calcium and phosphorous metabolism [6, 7]. Furthermore, we discovered that ClC-3 chloride channels are expressed by osteogenic lineage cells and regulate osteoblast differentiation. The level of ClC-3 chloride channels activation was found to have important impacts on the expression of osteogenesis-related genes, formation of mineralized nodules and acidification of the intracellular endosomal system in vitro [8, 9]. However, its regulatory mechanism is not well known. Transforming growth factor-β1 (TGF-β1) is a multidirectional, pleiotropic cytokine that participates in cell-surface receptor signaling and regulates cell proliferation in an autocrine or paracrine manner. It also plays a role in cell differentiation, apoptosis, bone formation and reconstruction [10, 11]. Our previous results suggest that ClC-3 chloride channels and TGF-β1 have an interesting relationship; that is, overexpression of ClC-3 can decrease the expression of TGF-β1, while decreased expression of ClC-3 chloride channels can increase TGF-β1 expression. Meanwhile, ClC-3 chloride channels and TGF-β1 exhibit colocalization in osteoblasts.

Based on the above context, TGF-β1 signaling was hypothesized to likely be involved in the functions of ClC-3 chloride channels in bone metabolism. Canonical TGF-β1 signaling can be mediated by Smads. Upon binding to TGF-β1 receptor (TβR) complexes, including type I and type II receptors, Smad2/3 is phosphorylated and activated, and Smad4 is then recruited to the Smad2/3 complex and translocated into the nucleus to direct the osteogenic gene transcriptional response [12–14]. In addition to Smads, TGF-β1 may promote the activation of mitogen-activated protein kinase (MAPK) signaling cascades in osteogenesis. Erk-MAPK and p38-MAPK are important signaling regulators of osteogenic differentiation and bone metabolism. Both pathways converge at Runx2, and the expression of Runx2 and Osterix (Osx) is firmly associated with Erk phosphorylation. In addition, Erk and p38 differentially mediate TGF-β1 and BMP functions in osteoblasts [15, 16].

Notably, there is crosstalk between the canonical and noncanonical TGF-β1 signaling pathways. The mechanisms of TGF-β1 signaling in bone metabolism are quite complex. Given the importance of TGF-β1 signaling in ClC-3 chloride channels, it is crucial to investigate the specific regulators of TGF-β1 signaling that participate in the regulatory effect of ClC-3 chloride channels on osteoblast differentiation in osteoblasts, as done in the current study.

## Materials and methods

### Cell culture and transfection

MC3T3-E1 cells, a mouse calvaria-derived preosteoblast cell line, were cultured in α-MEM (Gibco, USA) supplemented with 10% fetal bovine serum (FBS) (Gibco, USA), 1% penicillin–streptomycin and a 1% L-glutamine solution under 5% CO_2_ at 37 ℃. Before treatment, cells were plated on treated tissue culture plates and grown to 85% confluence. TβR inhibitor LY2109761 (Selleck, USA) was constituted in dimethyl sulfoxide and stored at − 80 °C. The cells were treated with LY2109761 at a concentration of 2 μM for 24 h to inhibit the expression of receptor complexes.

### RNA interference

MC3T3-E1 cells were transfected with a specific small interfering RNA (siRNA) and a control siRNA using Lipofectamine 2000 (Invitrogen, USA) according to the manufacturer’s instructions. The regular medium was replaced at 6 h, and the cells were continuously cultured for 48 h for the next step. The siRNA targeting mouse ClC-3 and Smad2/3/4 were synthesized by GenePharma company (Shanghai, China). The sense and antisense sequences were as follows, ClC-3: 5’-CGAGAGAAGUGUAAGGACATT-3’, 5’-UGUCCUUACACUUCUCUCGTT-3’; Smad2: 5’-UCUUUGUGCAGAGCCCCAA-3’, 5’-UUGGGGCUCUGCACAAAGA-3’; Smad3: 5’-GGAGAAAUGGUGCGAGAAG-3’, 5’-CUUCUCGCACCAUUUCUCC-3’; Smad4: 5’-GCCUGAUCUACACAAGAAUTT-3’, 5’-AUUCUUGUGUAGAUCAGGCTT-3’; negative control: 5’-UUCUCCGAACGUGUCACGUTT-3’, 5’-ACGUGACACGUUCGGAGAATT-3’.

### Quantitative real-time PCR

Total RNA was isolated from MC3T3-E1 cells using Trizol reagent (Invitrogen, USA), and cDNA was synthesized using PrimeScript™ RT Master Mix (Takara, Japan) by reverse transcription following the manufacturer’s instructions. Real-time PCR experiments were performed with the SYBR Green PCR kit (Takara, Japan) utilizing the ABI 7500 sequence detection system. The reaction system consisted of 2 μl of cDNA, 10 μl of SYBR® Premix Ex Taq, 0.8 μl of each PCR primer (forward and reverse), 0.4 μl of ROX and dH_2_O in a total volume of 20 μl. Then, the 2-ΔΔCt method was applied to calculate the relative changes in gene expression. The primer sequences used are shown in Table 1.

**Table 1:** The sequences of primers used in RT-PCR

Gene	Forward primer	Reverse primer
p38	5’-GCCCAAGCCCTTGCACAT-3’	5’-TGGTGGCACAAAGCTGATGAC-3’
Erk1	5’-GGTAGACGGTTCTGGAATGGAAGG-3’	5’-GTCAGGGAAAATGGGGTGGG-3’
Erk2	5’-TGTTCCCAAACGCTGACTCCA-3’	5’-AGTCGTCCAGCTCCATGTCAAACT-3’
Smad2	5’-TGTCGTCCATCTTGCCATTCACTC-3’	5’-TGTTCTCCACCACCTGCTCCTC-3’
Smad3	5’-CTTCACAGCCGTCCATGACAGTAG-3’	5’-CCAATGTAGTAGAGCCGCACACC-3’
Alp	5’-CCAACTCTTTTGTGCCAGAGA-3’	5’-GGCTACATTGGTGTTGAGCTTTT-3’
Runx2	5’-CGCCCCTCCCTGAACTCT-3’	5’-TGCCTGCCTGGGATCTGTA-3’
Osx	5’-ATGGCGTCCTCTCTGCTTG-3’	5’-TGAAAGGTCAGCGTATGGCTT-3’
Collagen I	5’-CGTGACCAAAAACCAAAAGTGC-3’	5’-GGGGTGGAGAAAGGAACAGAAA-3’
Gapdh	5’-CATGTTCCAGTATGACTCCACTC-3’	5’-GGCCTCACCCCATTTGATGT-3’

### Western blotting

As the protocol described, western blotting was performed with the following steps. Whole-cell lysates were prepared with lysis buffer (150 mmol/L NaCl, 50 mmol/L Tris–HCl, 100 mg/L PMSF, 1 mg/L aprotinin, 0.5% sodium deoxycholate, 1% SDS). The total protein concentration was determined via the BCA method, and proteins were transferred onto PVDF membranes. After blocking with milk (5% w/v) and incubating with p38, Erk1/2, Smad2, Smad3, Alp, Runx2, Osx, Collagen I or β‐actin (CST, USA) antibodies overnight at 4 ℃, the membranes were re‐probed with horseradish peroxidase‐labeled secondary antibodies. The HRP-ECL kit was used to enhance chemiluminescence in the blots, and XAR film exposure was applied to determine the densities of the bands.

### Immunocytochemistry

The cells were treated as described above and fixed with ice-cold 4% paraformaldehyde for 20 min, permeabilized with 0.5% Triton X-100 and 3% H_2_O_2_ in sequence, and blocked with 3% bovine serum albumin at 37 °C. Then, the cells were incubated with primary antibodies (CST, USA) overnight at 4 °C and conjugated secondary antibodies (CST, USA) for 45 min at 37 °C. Finally, the cells were incubated in a SABC wet box for 20 min, and the expression in each group was observed by a microscope.

### Coimmunoprecipitation assays

For coimmunoprecipitation (Co-IP) assays, protein lysates were prepared from MC3T3-E1 cells in precooled RIPA buffer containing protease inhibitors (ab156034). Cells were scraped into PBS and centrifuged for 15 min at 4 °C. The lysates were then incubated with antibody overnight and treated with protein A/G agarose beads to conjugate the antibody. The complex precipitate was washed with lysis buffer 3 ~ 4 times and resuspended in 2 × SDS loading buffer. The sample proteins were eluted from the agarose beads by immersion in boiling water at 95 °C for 5 min. The isolated proteins were measured by immunoblotting after separation by SDS–PAGE.

### Immunofluorescence analysis

MC3T3-E1 cells were plated in culture dishes specific for immunofluorescence at a density of 1 × 10^5^ cells/ml. Different groups of cells were treated accordingly, and then the cells were fixed with ice-cold 4% paraformaldehyde for 30 min, permeabilized with 0.25% Triton X-100 for 5 min, and blocked with 3% bovine serum albumin for 30 min at 37 °C. The cells were then incubated with primary antibodies (CST, USA) overnight at 4 °C, followed by secondary conjugated antibodies for 45 min at 37 °C after several rinses with PBS. Images were obtained using a laser scanning confocal microscope.

### Osteogenic differentiation and mineralization assays

MC3T3-E1 cells were separately transferred into six-well plates at a density of 1 × 10^4^ per well. Then, the cells were cultured for the corresponding number of days in osteogenic induction medium (10 mmol/L β‐glycerophosphate, 0.1 μmol/L dexamethasone and 50 μg/mL ascorbic acid). The induction medium was changed every 3 days.

After 7 days of induction, ALP activity was assessed by utilizing an ALP assay kit (Beyotime Biotechnology, Shanghai, China). The cell lysates were homogenized, and the supernatants were collected. Then, spectrophotometry was used to measure the absorbance at 405 nm to quantify ALP activity.

After 21 days of osteogenic induction, 2% Alizarin Red S staining (Sigma, USA) was applied for cell staining at 37 °C to evaluate mineralization of the cell matrix. An inverted microscope and camera system were used for further imaging. The concentration of Alizarin Red S dye emitted from the cell matrix into the cetylpyridinium chloride solution was determined by measuring absorbance at 540 nm on a spectrometer.

### Statistical analysis

All data were analyzed using SPSS statistical software (version 18.0; SPSS, Chicago, IL, USA) and are expressed as the mean ± standard deviation (SD). Statistical analysis was conducted using GraphPad Prism (version 6.00) for Windows. Student's t test was used to assess differences between two groups. Comparisons between three or more groups were analyzed by one-way analysis of variance (ANOVA) followed by the least significant difference (LSD) test and Student-Newman–Keuls (SNK) q test. Differences with a P value less than 0.05 were considered significantly different. All experiments were repeated more than three times.

## Results

### Effects of ClC-3 chloride channels on the expression of TGF-β1 signaling pathway proteins

**Fig. 1:**
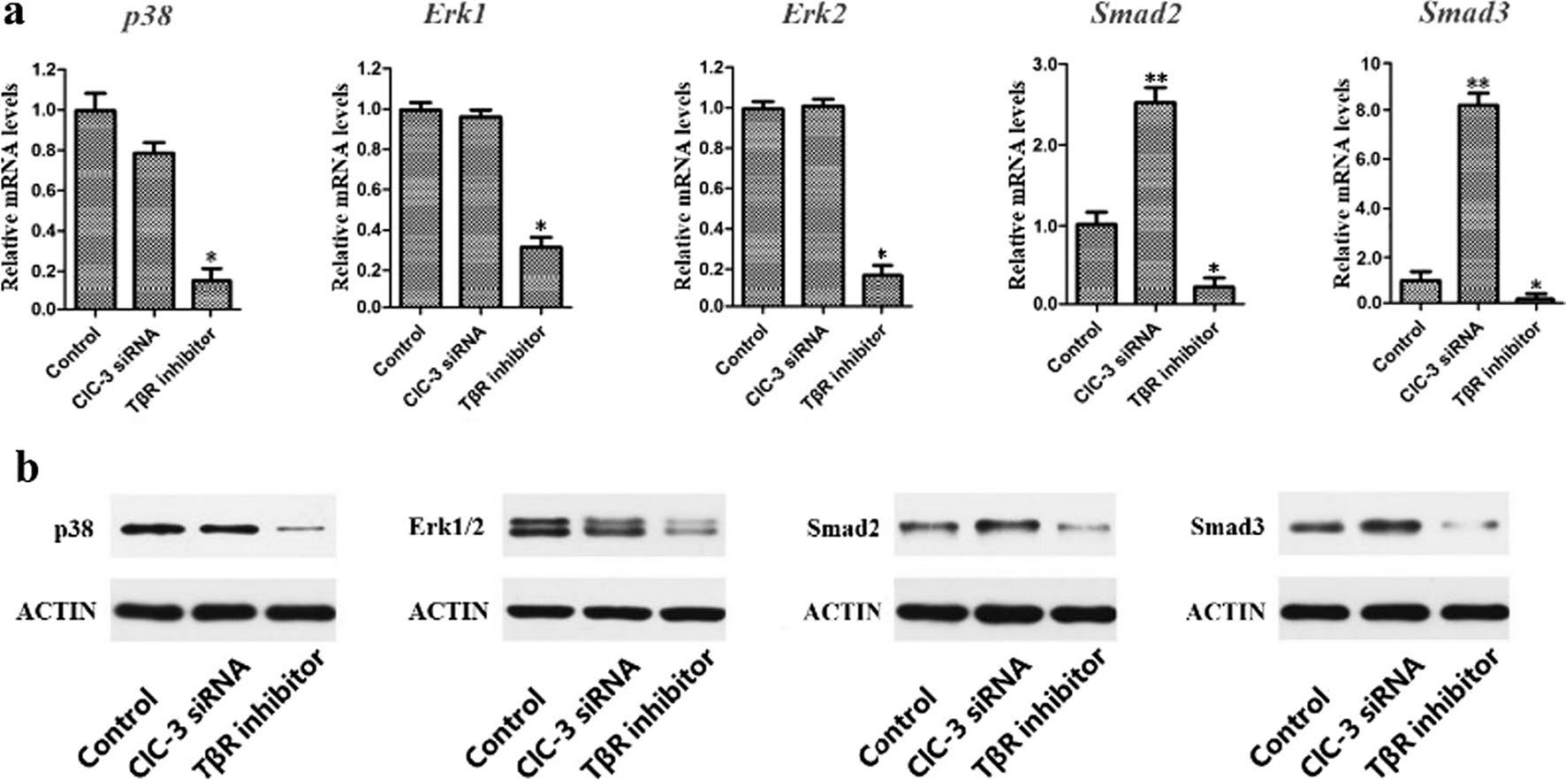
The effects of ClC-3 chloride channel and TβR knockdown on the expression of Smad and MAPK at both the mRNA (**a**) and protein (**b**) levels in MC3T3-E1 cells. The transcript and protein levels of Smad2, Smad3, p38, and Erk1/2 were significantly lower in the TβR knockdown group. Smad2 and Smad3 levels were higher in the ClC-3 knockdown group, while p38 and Erk1/2 levels were not significantly different at either the mRNA or protein level. *P < 0.05, **P < 0.01 compared with the control group

TGF-β1 signaling is mediated through the Smad-dependent pathway and the non-Smad-dependent pathway. To investigate the specific signal involved in the change in ClC-3 chloride channels, we selected Smad2 and Smad3 as target proteins of the Smad-dependent pathway and p38 and Erk1/2 as targets of the non-Smad-dependent pathway. Then, ClC-3 siRNA and TβR inhibitors were used to knock down the expression of ClC-3 chloride channels and TβR, respectively, and changes in the p38, Erk1/2, Smad2 and Smad3 genes and proteins were observed. The RT-PCR (Fig. 1a) and western blot (Fig. 1b) results showed that the expression levels of p38, Erk1/2, Smad2 and Smad3 in the TβR inhibitor group were lower than those in the control group. The expression levels of Smad2 and Smad3 in the ClC-3-knockdown group were increased compared with those in the control group, and the increase in Smad3 was greater than that in Smad2, while the expression levels of p38 and Erk1/2 were not significantly different between the two groups.

### Effects of ClC-3 chloride channels on the expression of Smad2 and Smad3 in the cytoplasm and nuclei of MC3T3-E1 cells

**Fig. 2:**
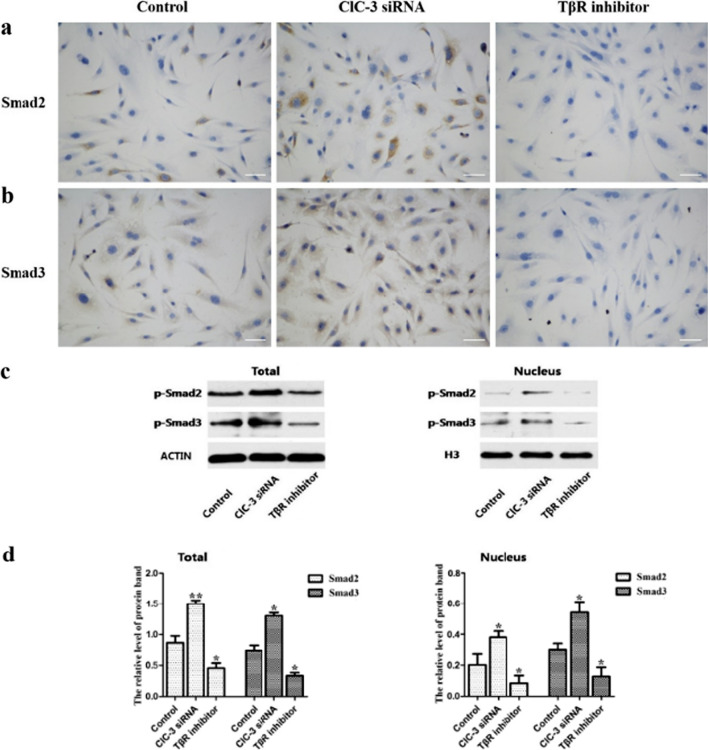
Knockdown of ClC-3 chloride channels promoted the expression of Smad2 and Smad3, while knockdown of TβR inhibited Smad2 and Smad3 expression in MC3T3-E1 cells. Immunocytochemical staining of Smad2 (**a**) and Smad3 in the three groups (**b**). The phosphorylation protein levels of Smad2 and Smad3 in the total proteins and nuclear proteins (**c**), and the relative quantification analysis diagrams of protein bands (**d**). Scale bars = 50 μm. *P < 0.05, **P < 0.01 compared with the control group

To analyze the difference in Smads levels in MC3T3-E1 cells, we detected the effects of ClC-3 chloride channels on Smad2 and Smad3 expression via immunocytochemistry. Compared with those in the control group, the Smad2 and Smad3 protein levels in the ClC-3 chloride channel knockdown group were higher, while those in the TβR inhibitor group were lower (Fig. 2a, b). Western blotting also demonstrated that the total Smad2 and Smad3 protein expression was increased in the ClC-3 siRNA group and decreased in the TβR inhibitor group compared with the control group. In addition, we isolated Smad2 and Smad3 proteins from the nucleus and found that the downregulation of ClC-3 chloride channels expression increased Smad2 and Smad3 nucleoprotein levels, while the inhibition of TβR expression decreased Smads nucleoprotein levels (Fig. 2c, d).

### Effects of ClC-3 chloride channels on Smad2/3/4 complex formation and translocation

**Fig. 3:**
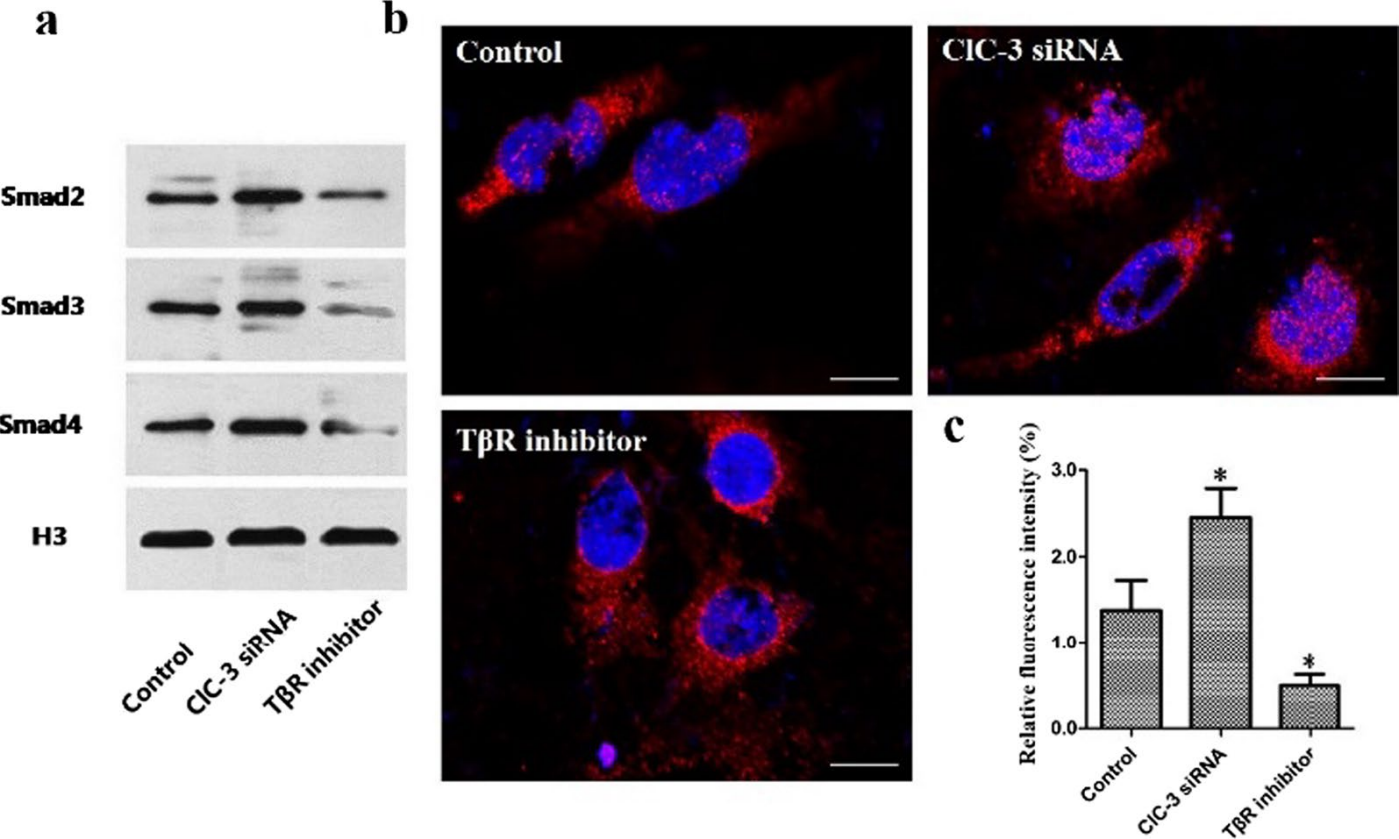
The effects of ClC-3 chloride channels on the nuclear translocation of Smad2/3/4. The formation of the Smad2/3/4 complex when ClC-3 and TβR were inhibited is shown (**a**), and the results were confirmed by quantitative densitometric analysis (**b**). The results of immunofluorescence analysis of Smad4 and quantification of the data are shown (**c**). Scale bars = 50 μm. *P < 0.05 compared with the control group

Smad2/3 binds Smad4 to form the Smad2/3/4 complex in the cytoplasm and enters the nucleus. To determine whether ClC-3 chloride channels expression is associated with formation of the Smad2/3/4 complex, Co-IP was carried out, and the data showed that ClC-3 knockdown promoted the Smad2/3/4 protein level compared to that in the control group, while knockdown of the expression of TβR inhibited the protein expression of Smad2/3/4 (Fig. 3a). To further detect the effects of ClC-3 chloride channels on the nuclear translocation of the complex, we chose the Smad4 protein as a representative component of the complex, and the labeled protein was observed with laser confocal microscopy. The results indicated that Smad4 was expressed in the cytoplasm and nucleus in all three groups. Furthermore, the fluorescence signal of Smad4 in the ClC-3 siRNA group was more intense than that in the TβR inhibitor group, while the intensity in the control group was somewhere in between (Fig. 3c). The Smad4 distribution in the ClC-3 siRNA group was wide and uniform, but in the TβR inhibitor group, the Smad4 distribution was sparse (Fig. 3b).

### Effects of the Smad2/3/4 pathway on the regulatory effect of ClC-3 chloride channels on the osteogenic differentiation in MC3T3-E1 cells

**Fig. 4:**
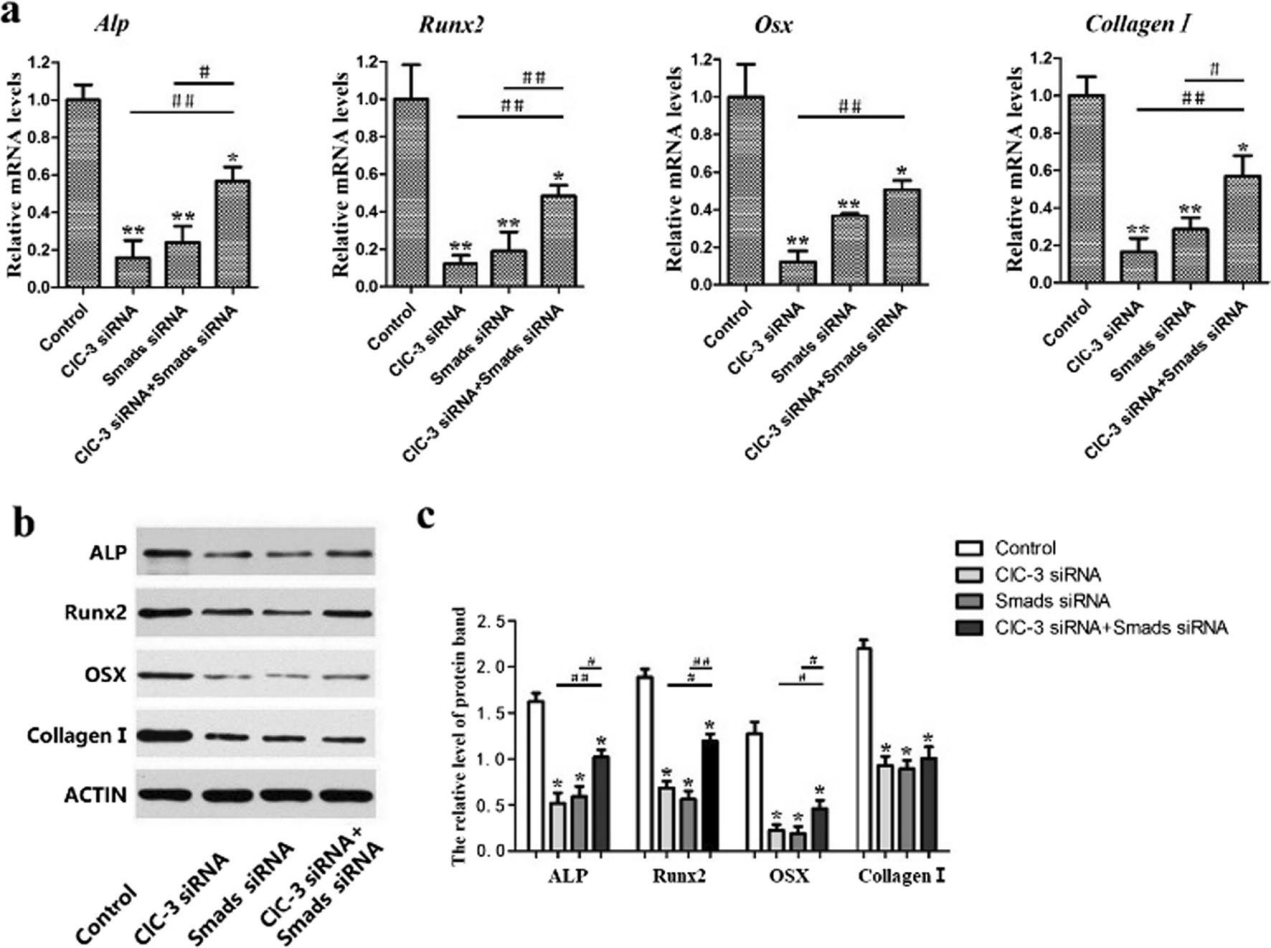
The effects of the Smad2/3/4 signaling pathway on ClC-3-mediated regulation of osteogenic genes and proteins in MC3T3-E1 cells. The mRNA levels of Alp, Runx2, Osx and Collagen I were significantly decreased when ClC-3 and Smads were silenced, but the degree of this decrease was lower when ClC-3 and Smads were silenced together than in the two former groups (**a**), as was protein expression of ALP, Runx2, Osx and Collagen I (**b**), and the relative quantification of protein bands (**c**). *P < 0.05, **P < 0.01 compared with the control group. ^#^*P* < 0.05, ^##^*P* < 0.01 compared between the experimental groups

ClC-3 chloride channels are expressed in osteogenic lineage cells and regulate osteoblast differentiation, and the expression level of ClC-3 chloride channels is closely related to the expression of osteogenesis-related genes, the formation of mineralized nodules in vitro and the acidification of the intracellular endosomal system. To investigate whether the Smad2/3/4 signaling pathway is involved in the regulatory effect of ClC-3 chloride channels on osteoblast differentiation, we examined the expression of osteogenesis-related markers at the gene and protein levels. The real-time PCR results showed that the osteogenesis-related genes (Alp, Runx2, Osx and Collagen I,) in the ClC-3 siRNA group, Smads siRNA group and ClC-3 + Smad siRNA group were lower than those in the control group. Runx2 and Osx levels decreased by 80% and 90%, respectively, and Alp levels decreased by 70% when ClC-3 was expressed at low levels. However, the expression of osteogenic genes in the ClC-3 + Smads siRNA group was lower than that in the control group but higher than that in the ClC-3 siRNA group and Smads siRNA group, but there was no significant difference in osteogenic gene expression between the ClC-3 siRNA group and Smads siRNA group (Fig. 4a). The protein expression levels of Alp, Runx2 and Osx followed essentially the same trend reveled by the real-time PCR data, except for Collagen I. The western blot data revealed that the protein expression of Collagen I remained steadily lower in the ClC-3 siRNA group, Smads siRNA group and ClC-3 + Smads siRNA group when compared with the control group, and Collagen I protein expression did not differ significantly among the three experimental groups (Fig. 4b, c).

**Fig. 5:**
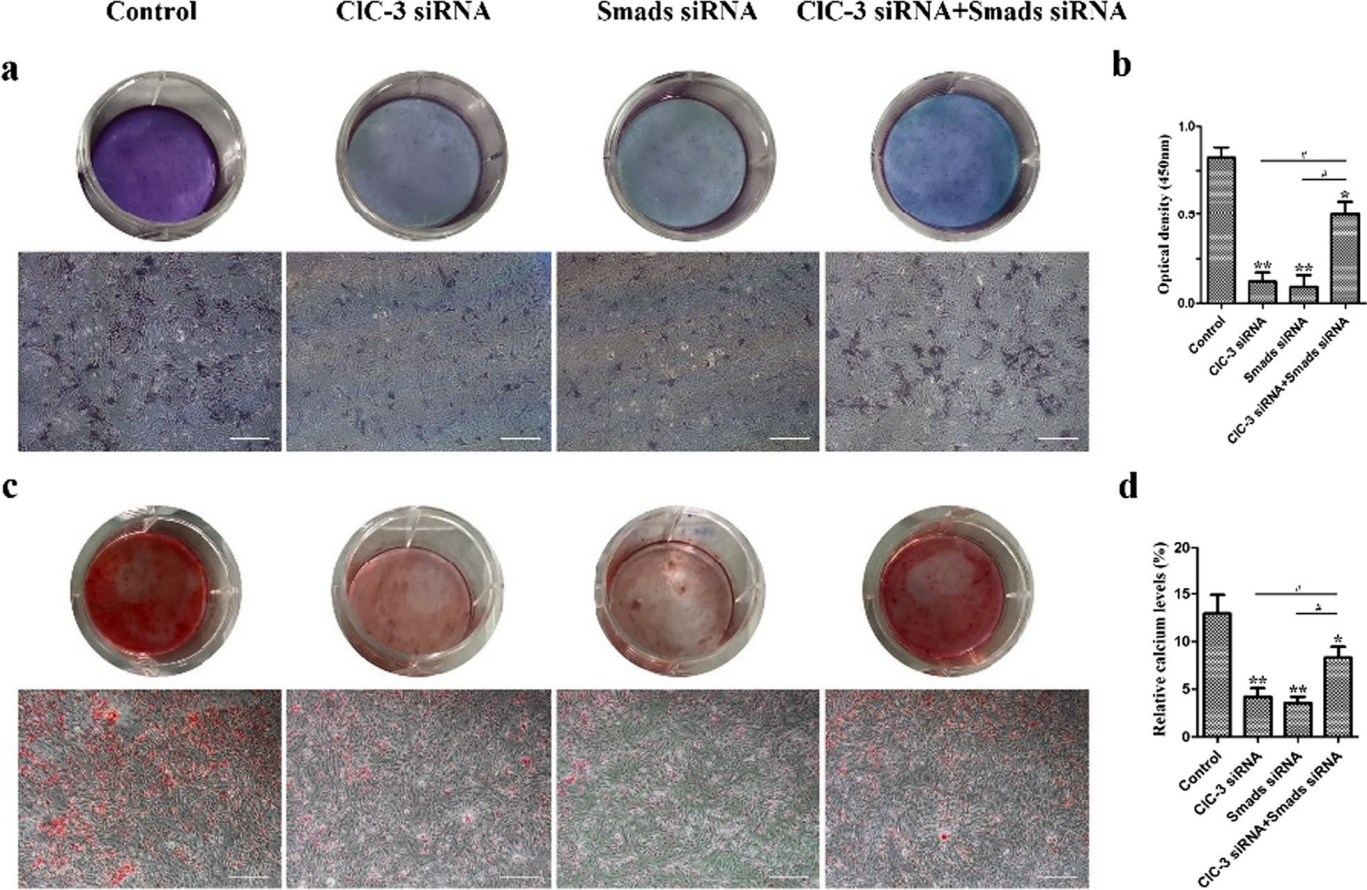
The Smad2/3/4 signaling pathway plays a negative role in ClC-3 chloride channel-mediated regulation of osteogenic differentiation in MC3T3-E1 cells. Osteogenesis was determined by ALP staining (**a**) and ALP activity assays (**b**). Alizarin Red S staining (**c**) and an assessment of calcium deposition (d) were carried out. Scale bars = 100 μm. *P < 0.05, **P < 0.01 compared with the control group. ^#^*P* < 0.05, ^##^*P* < 0.01 compared between the experimental groups

ALP activity is considered an earlier marker that reflects the mature state of osteoblasts, and mineralized extracellular matrix nodules are a sign of mature osteoblasts. We then detected the ALP activity of MC3T3-E1 cells 7 days after osteogenic induction and found that the activity level in the control group was the highest, followed by that in the ClC-3 + Smads siRNA group, the ClC-3 siRNA group and the Smads siRNA group (Fig. 5a, b). Twenty-one days after osteogenic induction, red calcium nodules were detected through alizarin red s staining and quantified at by determining the OD value at 450 nm. The results showed that the knockdown of ClC-3 and Smads protein expression decreased the amount of calcium nodules. In addition, the reductions when ClC-3 or Smads proteins were individually knocked down were significantly greater than those upon knockdown of ClC-3 and Smads together (Fig. 5c, d).

## Discussion

Bone metabolism is the result of balance between bone resorption and bone formation, and stable bone regulation is the basis for the normal maintenance of biological activities. Ion channel proteins, hormones and many other cytokines participate in the regulation of bone remodeling [17, 18]. ClC-3 chloride channels, which are widely expressed in osteoblasts, play an important role in osteoblast differentiation. In a further study on the related mechanism, the regulation of ClC-3 chloride channels was shown by the data to be closely associated with TGF-β1. TGF-β1 is initially produced by osteoblasts and exists in an inactive form; then, it is activated by the acidic bone microenvironment and regulates bone remodeling. The Smad and MAPK signaling pathways are both mediated by TGF-β1; however, which pathway is involved in the regulation of osteogenic differentiation by ClC-3 chloride channels was not clear. Therefore, our current study aimed to demonstrate the specific role of the TGF-β1 signaling pathway in the effect of ClC-3 chloride channels on osteoblasts.

In general, both the Smad and MAPK pathways are activated through TGF-β1 induction during osteoblast differentiation, which is essential for the formation of the skeleton [19–22]. To further evaluate the specific pathway involved in ClC-3 chloride channels-mediated regulation of osteogenic differentiation, we assessed the roles of Smad2, Smad3, p38 and Erk in MC3T3-E1 cells. Our results demonstrated that when the expression of ClC-3 chloride channels was inhibited, the expression of the p38 and Erk1/2 proteins did not change significantly compared with that in the control group, while the expression of the p38 and Erk1/2 proteins was decreased when a TβR inhibitor was used. This finding indicates that activation of the signaling proteins p38 and Erk1/2 can be achieved via the binding of TGF-β1 and its receptors. Disruption of this binding will affect Erk1/2 and p38-MAPK pathway signal transduction, thus potentially affecting the corresponding biological functions regulated by TGF-β1. However, Erk1/2 and p38 were not significantly correlated with the change in ClC-3 levels, which suggests that the MAPK pathway is likely not involved in the regulatory effect of ClC-3 chloride channels on osteogenic differentiation. Nevertheless, the expression of Smad2 and Smad3 was decreased in the TβR inhibitor group but increased in the ClC-3 siRNA group, suggesting that the activation of Smad2 and Smad3, similar to that of Erk1/2 and p38, was required for the binding of TGF-β1 and its receptors, which is consistent with the results of previous studies [23, 24]. However, ClC-3 knockdown promoted the expression of Smad2 and Smad3, which may be because ClC-3 chloride channels promoted the expression of TGF-β1, thus indirectly promoting Smad2 and Smad3 protein expression. Based on the above results, we speculated that the Smad2/3 signaling pathway can respond to a change in ClC-3 chloride channels in MC3T3-E1 cells.

Subsequently, we further investigated the nuclear translocation of the Smad complex when ClC-3 chloride channels were expressed at different levels. Phosphorylation of Smad2/3 is a key step and marker in the Smad signaling pathway [25, 26]. Our data showed that the expression of phosphorylated Smad3 increased by 44.4% and 45.1% among the total protein and nuclear protein, respectively, in the ClC-3 siRNA group, while phosphorylated Smad2 levels increased by 39.8% and 41%; additionally, the Smad3 level decreased to a greater extent than the Smad2 level in the TβR inhibitor group. This result indicated that ClC-3 knockdown promoted the nuclear translocation of Smad2/3 and that Smad3 is more sensitive to ClC-3 chloride channel stimulation, which is consistent with the immunocytochemistry results. This may be related to the direct binding of Smad3 to TGF-β1 receptors and subsequent regulation of the transcription of many effector genes [27, 28]. Similarly, low ClC-3 expression increased the formation of the smad2/3/4 complex, while inhibition of the receptors had the opposite effect. This further suggests that the Smad signaling pathway participates in the functions of the ClC-3 chloride channels. Furthermore, we detected the change in intracellular Smad4 localization using immunofluorescence staining. Studies have shown that CD147 plays a key role in the translocation of Smad4 from the cytoplasm to the nucleus, and silencing CD147 gene expression promoted Smad4 nuclear translocation [29, 30]. However, whether CD147 is related to the functions of ClC-3 chloride channels requires further exploration.

Since Smad2/3/4 are closely related to the expression of ClC-3 chloride channels, it is particularly important to determine the mechanism by which the Smad2/3/4 signaling pathway regulates ClC-3 chloride channels-mediated regulation of osteogenic differentiation. We knocked down the ClC-3 chloride channels and Smad2/3/4 complex and detected the expression of osteogenic genes and the formation of mineralized nodules. ALP activity is an early marker of osteogenic differentiation, and ALP is highly active in the early stage of bone formation. Runx2 is a key transcriptional regulator that acts as a “switch” to influence the bone formation marker Osx [31–34]. In general, Collagen I is involved in organ development, wound healing and tissue repair through its interactions with growth factors and cytokines, and Collagen I is more sensitive, specific and stable than other markers [35, 36]. Our data indicated that ALP activity and Collagen I, Runx2 and Osx levels were decreased in both the ClC-3 and Smads siRNA groups. Runx2 and Osx levels were decreased by 80% and 90%, respectively, and ALP activity was decreased by 70% when ClC-3 chloride channels was expressed at low levels. Although the expression levels of genes and proteins were inconsistent, the overall trends were the same. This suggests that ClC-3 chloride channels begin to have an effect at the early stage of osteogenic differentiation but play an important role in the late stage of osteogenic differentiation. Similarly, when we inhibited the Smad2/3/4 signaling pathway, the expression of osteogenesis markers was significantly decreased, which also suggests that the activation of Smads can promote osteogenic differentiation. Notably, when we knocked down the expression of ClC-3 and Smad2/3/4 together, we found that ALP activity and Runx2 and Osx levels were also decreased, but the extent was less pronounced than the effects of their inhibition alone, except for the Collagen I protein. This is probably related to the fact that Collagen I is a major component of the matrix and regulates the adhesion and bone-guiding function of mature osteoblasts after differentiation,; nevertheless, the ClC-3 chloride channel is mainly involved in regulating the osteoblast differentiation process [7, 37].

In addition, we continued to detect ALP activity and calcium nodule formation after osteogenic induction in MC3T3-E1 cells [38, 39], and the trends were consistent with those of osteogenic gene expression. Quantitative analysis of calcium node formation a greater reduction than the reduction in ALP activity, which further demonstrated the main regulatory role of ClC-3 chloride channels in the late stage of osteogenic differentiation. Moreover, the effect of knocking down ClC-3 chloride channels and Smad2/3/4 together was less pronounced than the effects in the ClC-3 or Smad2/3/4 siRNA groups. Collectively, these data suggest that the Smad2/3/4 signaling pathway is involved in the regulatory effect of ClC-3 chloride channels on osteoblast differentiation, and this pathway is likely to play a negative regulatory role. Certainly, there must be other signaling pathways that promote the role of ClC-3 chloride channels in regulating osteoblasts that need to be further explored [40].

## Conclusions

ClC-3 chloride channels affect the regulation of osteogenic differentiation, and Smad signaling can respond to ClC-3 chloride channels. The expression of ClC-3 chloride channels decreased the formation of Smad2/3/4 and the ability of the complex to translocate to the nucleus. Inhibition of Smad2/3/4 abrogated the inhibition of osteogenic markers when ClC-3 chloride channels were knocked down. In conclusion, the Smad2/3/4 signaling pathway may act as a negative regulator of osteogenic differentiation regulation by ClC-3 chloride channels in MC3T3-E1 cells.

## Data Availability

Data will be available upon request by the first author.
